# The impact of prenatal maternal mental health during the COVID-19 pandemic on birth outcomes: A cohort study within the CONCEPTION cohort

**DOI:** 10.1192/j.eurpsy.2023.741

**Published:** 2023-07-19

**Authors:** A. Berard, V. Tchuente, N. Pages, J. Gorgui, T. Fareh, S. King, G. Elgbeili

**Affiliations:** 1 Faculty of Pharmacy, University of Montreal; 2 Research Center, CHU Sainte-Justine, Montreal, Canada; 3 Faculty of Medicine, Université Claude Bernard Lyon 1, Lyon, France; 4 Research Center, CHU Sainte-Justine; 5 Research Center, CHU Sainte-Justine, Montréal; 6 Faculty of Medicine, Université Claude Bernard Lyon 1, Lyon; 7 Faculty of Medicine, McGill University, Montreal, Canada

## Abstract

**Introduction:**

External natural events, such as the COVID-19 pandemic, can contribute to increased stress, depression and anxiety in pregnant persons. Thus far, studies on the impact of maternal mental health during the pandemic on perinatal outcomes have been conflicting.

**Objectives:**

Assess the impact of prenatal mental health during the COVID-19 pandemic on preterm birth (PTB) and low birthweight (LBW).

**Methods:**

Pregnant individuals, >18 years were recruited in Canada, their data were collected through a web-based questionnaire. Our analysis includes data on individuals recruited between 06/2020 and 08/2021, who completed questionnaires at baseline and 2-month post-partum. Data on maternal sociodemographic, comorbidities, medication, mental health measures (Edinburgh Perinatal Depression Scale, General Anxiety Disorder-7, stress), hardship (CONCEPTION study Assessment of Stress from COVID-19 –150 points), gestational age at delivery and birth weight were self-reported. PTB defined as delivery before 37 weeks of gestation. LBW defined as birth weight less than 2,500 grams.

**Results:**

A total of 1,265 and 1,233 participants were included in the analyses of PTB and LBW, respectively. After adjusting for potential confounders, we found no differences between prenatal mental health and PTB ([depression [adjusted RR [aRR] 1.01, CI 95% 0.91 to 1.11], anxiety [aRR 1.04, CI 95% 0.93 to 1.17], stress [aRR 0.88, CI 95% 0.71 to 1.10], hardship [aRR 1.00, CI 95% 0.96 to 1.04]). However, we found that the risk of PTB was increased with ethnicity/race (aRR 3.85, CI 95% 1.35 to 11.00), obstetrician/gynecologist follow-up (aRR 2.77, CI 95% 1.12 to 6.83). We didn’t find any significant association between prenatal mental health and LBW. However, annual household income, previous delivery were associated with a decreased risk of LBW (aRR 0.15, CI 95% 0.05 to 0.49; aRR 0.39, CI 95% 0.20 to 0.77, respectively).

**Conclusions:**

Conclusion: No association was found between prenatal mental health during the COVID-19 pandemic and the risk of PTB or LBW. However, it is imperative to continue the follow-up of mothers and their offspring in order to detect early any long-term health problems.

**Disclosure of Interest:**

None Declared

